# 

**Published:** 1892-02-27

**Authors:** 


PRICE ONE PENNY.

283, Vol. XI.—February 27th, 1892.

[Registered at the General Post Office as a Newspaper for transmission in the United Kingdom and Abroad.]

WITH EXTRA NURSING SUPPLEMENT.
Per Annum, 6s. 6d., post free.
Monthly Parts, 6d.; post free, 8d.
Ditto per Annum, 8s., post free.

### THE HOSPITAL

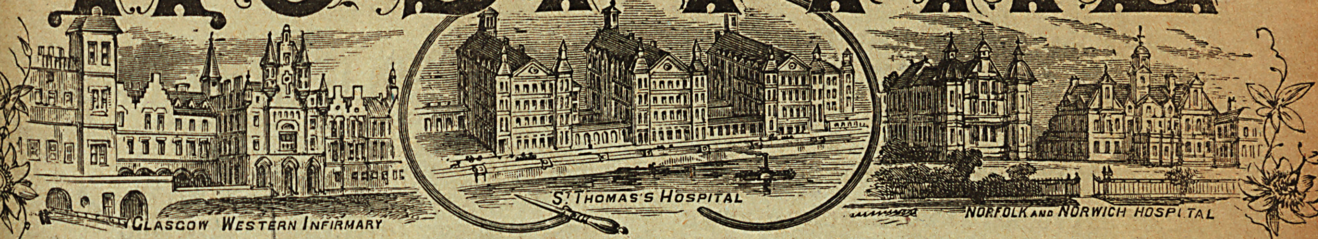

WITH EXTRA NURSING SUPPLEMENT

No. 283. Vol. XI.] SATURDAY, FEBRUARY 27th, 1892. [ONE PENNY.

